# Mediastinal extraosseous chordoma in a teenager: Diagnosis by ultrasound-guided percutaneous biopsy

**DOI:** 10.1016/j.radcr.2022.07.085

**Published:** 2022-08-10

**Authors:** Amir-Ali Mahmoud, Eric T. Wei, Kiyon Naser-Tavakolian, Amit Gupta

**Affiliations:** a Stony Brook Renaissance School of Medicine, 100 Nicolls Rd, Stony Brook, NY 11794, USA; b Stony Brook University Hospital, Department of Radiology, 101 Nicolls Rd level 4, Stony Brook, NY 11794, USA

**Keywords:** Pediatric, Thoracic, Chordoma, Ultrasound, Percutaneous, Biopsy

## Abstract

Pediatric chordomas are rarely described in the literature with most cases being managed surgically followed by adjuvant radiotherapy for local control. We present a case of an 18-year-old female with thoracic chordoma causing significant mass effect resulting in tracheal deviation, esophageal compression, and splaying of the great vessels. Ultrasound-guided anterior left transcervical percutaneous biopsy of the neck with surgical pathology immunohistochemistry confirmed the presence of chordoma. The patient underwent extensive palliative debulking followed by radiation therapy leading to clinical improvement. This case demonstrated that an ultrasound-guided percutaneous biopsy is an essential procedure in the diagnosis and treatment of chordoma, which led to successful treatment when followed by surgery and radiation.

## Introduction

Chordomas are rare locally invasive malignant tumors arising from notochord remnants that are even rarer in the pediatric population [1]. Approximately 94% of chordomas present in the spinal, sacrococcygeal or cranial region, and few pediatric reports have been documented in the literature of thoracic chordomas in the pediatric population. Chordomas are classically thought to be intraosseous lesions, however, extraosseous chordomas are an even more variant. We present a case of an 18-year-old female with an extraosseous thoracic chordoma involving the mediastinum and review the pertinent imaging findings.

## Case report

An 18-year-old female, with history of asthma, presented to the emergency department for progressively worsening shortness of breath and dysphagia. The patient's symptoms began approximately 18 months prior to presentation initially with intermittent mild shortness of breath that slowly progressed. Her pediatrician ordered a chest radiograph that demonstrated the presence of a small mediastinal mass. A follow-up radiograph reportedly showed resolution of the mass a year before her current presentation. The patient followed up with cardiology with a reportedly normal EKG and echocardiogram. Because her symptoms persisted, she was referred to a pulmonologist and subsequently diagnosed with moderate-persistent asthma and prescribed inhaled corticosteroids and albuterol. A computed tomography (CT) chest was also recommended to evaluate for the mediastinal mass but was never obtained. The patient's symptoms continued to worsen, and she experienced chest pain and dyspnea with exertion, dysphagia to solids and liquids, and intermittent left arm numbness. A repeat chest radiograph just before the current presentation demonstrated interval enlargement of the anterior mediastinal mass, and the patient was referred to the emergency department for further evaluation.

**Fig. 1:**
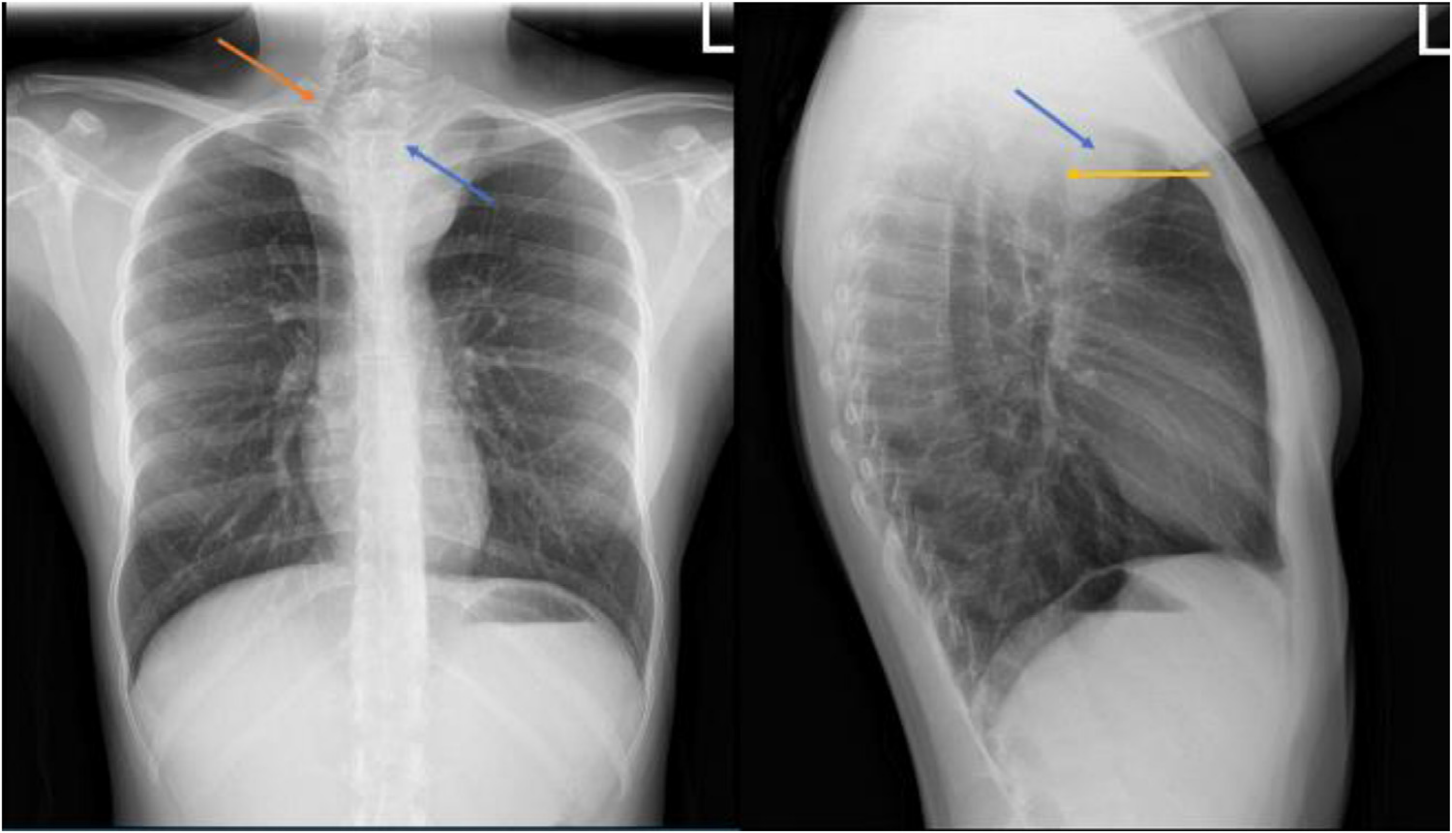
Upright frontal and lateral radiographs demonstrate soft tissue mass (blue arrow) projecting over the mediastinum causing left to right shift of the trachea (orange arrow) with focal stenosis (yellow arrow). (Color version of figure is available online.)

**Fig. 2:**
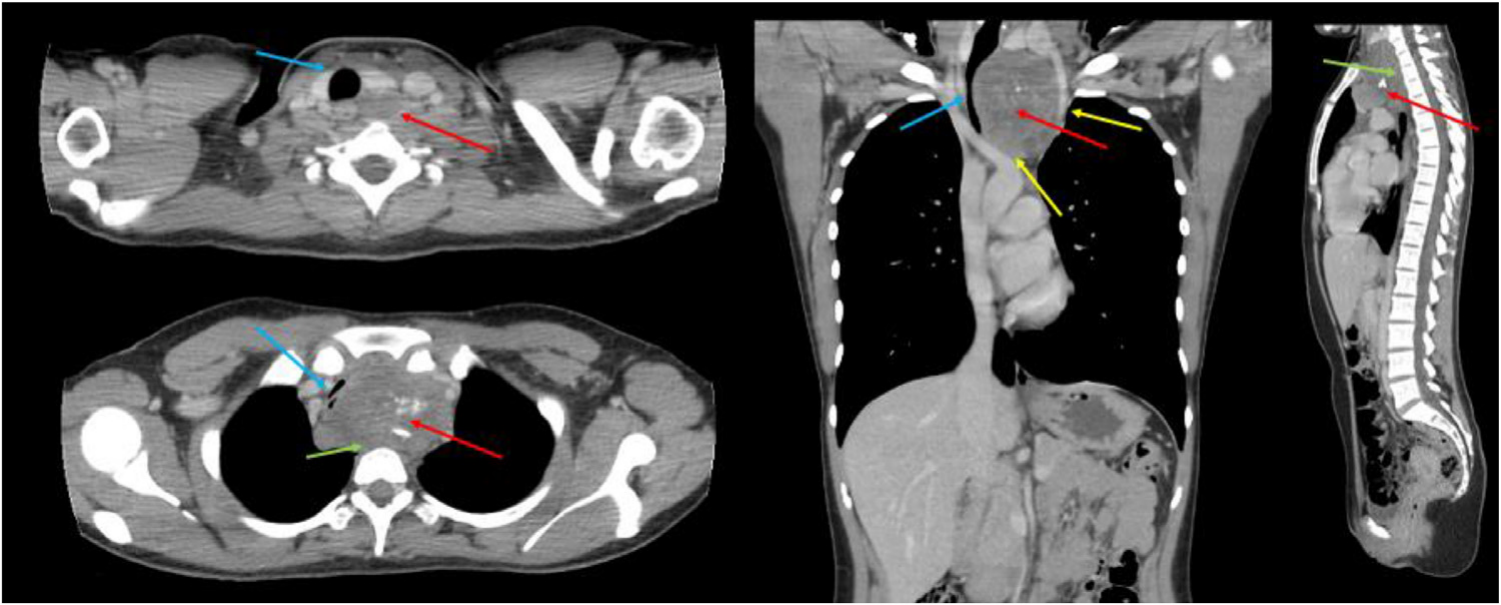
Contrast enhanced axial (top and bottom left), sagittal (right), and coronal (middle) CT images demonstrates heterogenous upper mediastinal mass with internal calcifications (red arrows) that measured approximately 7.5 × 7.4 × 7.6 cm interposed between and splaying the great vessels (yellow arrows). This lesion extends superiorly and inferiorly causing severe left to right mass effect and compression on the trachea (blue arrows). There is abutment of the lesion on the cervicothoracic spine (green arrow). (Color version of figure is available online.)

**Fig. 3:**
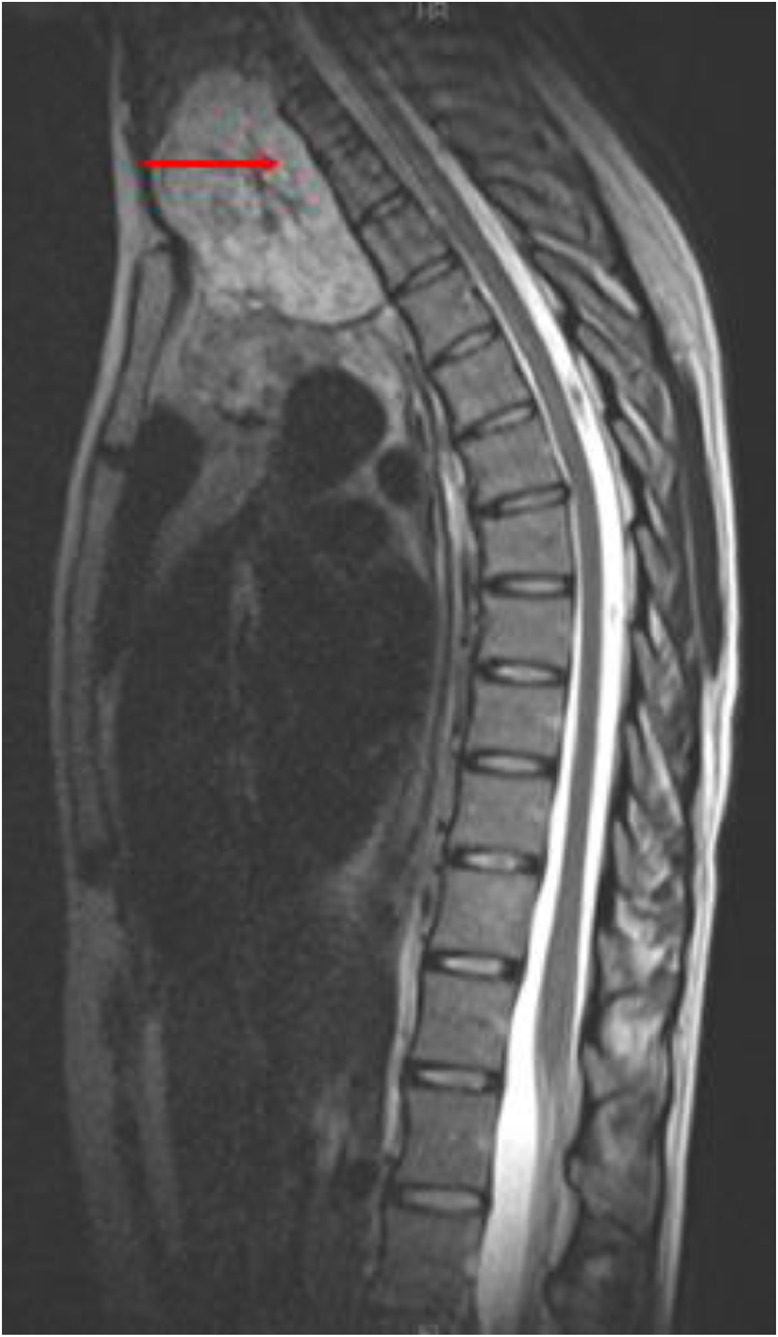
MRI Spine demonstrates heterogenous T2 lesion with enhancement in the upper mediastinum (red arrow) that abuts the ventral surface of the cervical spine without signal abnormalities of the vertebral body. (Color version of figure is available online.)

Chest radiographs in the emergency department demonstrated a large superior mediastinal mass with significant rightward deviation of the trachea (Fig. 1). A CT chest showed a 7.5 × 7.4 × 7.6 cm heterogeneous mass with internal calcifications that extended from the left lower neck and into the upper anterior mediastinum causing significant mass effect and compression of the trachea, esophagus, and splayed the great vessels (Fig. 2). Magnetic resonance imaging of the thoracic spine showed abutment of the lower cervical and upper thoracic vertebral bodies' ventral surface without definite CT evidence of invasion into the vertebral bodies or spinal canal (Fig. 3).

**Fig. 4:**
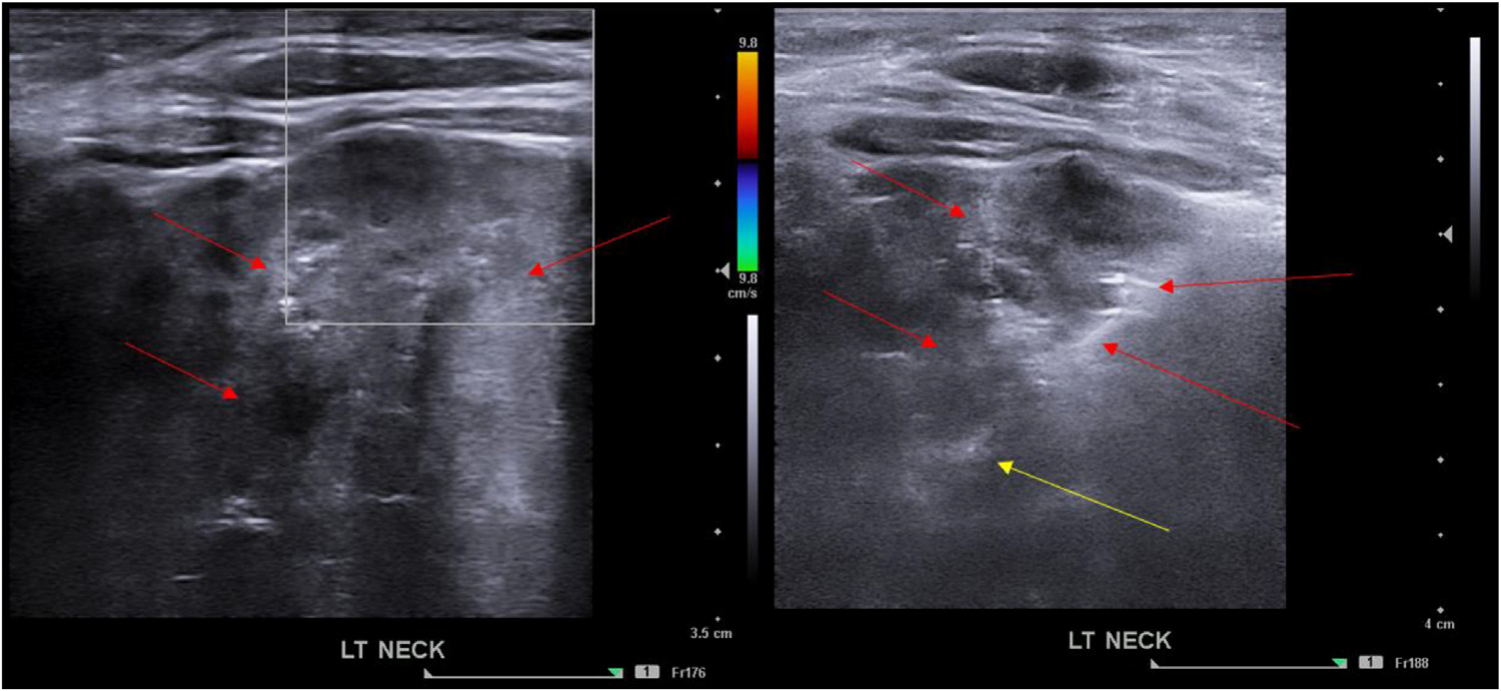
Ultrasound guided core needle (yellow arrow) biopsy from the left anterior neck approach of the heterogeneous mediastinal mass. (Color version of figure is available online.)

Interventional Radiology was consulted for percutaneous biopsy. An ultrasound-guided anterior left transcervical neck approach biopsy with a 17-gauge introducer needle and an 18-gauge core biopsy needle was performed with cytology present; 8 cores were obtained (Fig. 4). Surgical pathology with Brachyury immunohistochemistry confirmed chordoma.

The patient underwent extensive palliative debulking operation with pediatric surgery, cardiothoracic surgery, and orthopedic surgery in an inherently high-risk surgery in this patient due to the location of the lesion and because the airway was 90% collapsed. At surgery, primary median sternotomy was performed, and the mediastinal mass was found to originate from above the aortic arch and extended up to the base of the neck. There was compression of the trachea and esophagus with rightward deviation and splayed the takeoff of the great vessels. The lesion was carefully dissected off the innominate vein, innominate artery, thyroid, trachea, esophagus, left carotid artery, left internal jugular, aortic arch, and cervicothoracic spine. Small remnants remained on the esophagus due to concern of mucosal rent. The patient tolerated the surgery well and recovered in the cardiothoracic ICU. Surgical pathology also demonstrated an area of focal invasion through a fragment of cortical bone of a vertebral body. The remainder of her stay was complicated by chylothorax, right ulnar nerve neuritis, and vocal cord palsy. She was followed outpatient for radiation therapy.

## Discussion

Chordomas are rare, slow-growing malignant bone tumors of notochord origin or ectopic notochordal rests with an incidence of 0.08 per 100,000 persons. They have a high local recurrence and have a peak incidence between 50 and 60 years of age. Additionally, they predominate in adult men compared to women [2]. Chordomas are rare in patients less than 40 years of age and rarely affect children, which only accounts for less than 5% of cases [1]. Cranial, spinal, and sacral chordomas comprise 94% of all lesions in adults. Similarly, in children, the most common sites are sacrococcygeal and cranial (spheno-occipital). There are few case reports of thoracic chordomas in the pediatric population, most recently described by Soudack et al. in 2006. Their report described including their case, pediatric thoracic chordomas to date [1,3]. Thoracic chordomas will present depending on the direction of their growth, specifically, whether they grow anteriorly towards the mediastinal structures or posteriorly towards the spinal canal. Soudack et al. found no preference for a specific age, sex, or growth pattern of the tumors in the prior cases.

The typical appearance of a chordoma on radiography depends on its location. In radiography, intraosseous chordomas will appear as a destructive lytic mass of the sacrum or arising from a vertebral body if spinal and may enlarge the neural foramina. They may have internal calcification depending on the degree of matrix present, but this is more common in the clival chondroma. There is often an associated soft tissue mass in the prevertebral location, but it may extend into the epidural space. On CT and magnetic resonance imaging, chordomas will be a centrally located, destructive lytic lesion with an expansile soft tissue mass which may have internal necrosis, will be intermediate to low signal on T1, very high signal on T2, and demonstrate moderate to marked enhancement depending on the degree of internal necrosis/hemorrhage [4,5]. Extraosseous chordomas, in contrast to intraosseous chordomas, present typically as discrete soft tissue lesions and are even rarer than intraosseous chordomas and tend to have less bony involvement, as in our presented case. The etiology of the extraosseous notochord remnant has not been elucidated, however, can be conjectured to be migratory or ectopic in nature [6].

A posterior mediastinal mass in a child has a broad differential. Due to the presence of many tissue elements, neurogenic tumors, neurogenic, bronchial, and foregut cysts, lymphadenopathy, and extramedullary hematopoiesis are considerations. Again, chordomas in the thoracic region are quite rare and, in a patient under the age of 40, are even rarer [3]. In our case, we noted the chordoma to have extended anteriorly and caused significant compression of the upper mediastinal structures, including the trachea and great vessels. The degree of mass effect and displacement was atypical compared to prior case reports. The size of the mass in our patient was significantly larger at presentation and abutted multiple anterior and middle mediastinal structures. Only one reported case (Huang et al.) described anterior tracheal deviation, with the remainder mainly having vertebral involvement with local mass effect on the mediastinum [3,7,8].

Typical management of a chordoma is surgical, with the entire tumor, including its capsule, being excised. Adjuvant radiotherapy is recommended in most cases as it is shown to improve local control. However, due to chordomas' sensitive location and surrounding structures, this often leads to dose-limiting therapy [9]. However, the prognosis is poor for children with chordomas due to their high rates of recurrence. Because chordomas are rare in the pediatric population, more extensive studies haven't been performed to evaluate survival after resection. However, in adults, the overall 10-year survival is approximately 40% due to these tumors' locally aggressive nature [10].

## Conclusion

We report a case of successful ultrasound-guided percutaneous biopsy of a chordoma in an 18-year-old female. More follow up is needed to determine the likelihood of recurrence of the chordoma and evaluate survival after resection.

## Patient consent statement

Informed consent was obtained from this patient prior to submitting this manuscript.

## References

[1] Coffin CM, Swanson PE, Wick MR (1993). Chordoma in childhood and adolescence. A clinicopathologic analysis of 12cases. Arch Pathol Lab Med.

[2] Walcott BP, Nahed BV, Mohyeldin A, Coumans JV, Kahle KT, Ferreira MJ. (2012). Chordoma: current concepts, management, and future directions. Lancet Oncol.

[3] Soudack M, Guralnik L, Ben-Nun A, Berkowitz D, Postovsky S, Vlodavsky E, Engel A (2007). Imaging features of posterior mediastinal chordoma in a child. Pediatr Radiol.

[4] Murphey MD, Andrews CL, Flemming DJ, Temple HT, Smith WS, Smirniotopoulos JG. (1996). From the archives of the AFIP. Primary tumors of the spine: radiologic pathologic correlation. Radiographics.

[5] Smolders D, Wang X, Drevelengas A, Vanhoenacker F, De Schepper AM (2004). Value of MRI in the diagnosis of non-clival, non-sacral chordoma. Skelet Radiol.

[6] DiFrancesco LM, Davanzo Castillo CA, Temple WJ (2006). Extra-axial chordoma. Arch Pathol Lab Med.

[7] Ahrendt MN, Wesselhoeft CW (1992). Chordoma presenting as a posterior mediastinal mass in a pediatric patient. J Pediatr Surg.

[8] Huang SM, Chen CC, Chiu PC, Lai PH, Ho JT, Tseng HH (2003). Unusual presentation of posterior mediastinal chordoma in a 2-year-old boy. J Pediatr Hematol Oncol.

[9] De Amorim Bernstein K, DeLaney T. (2016). Chordomas and chondrosarcomas–the role of radiation therapy. J Surg Oncol.

[10] Zou MX, Lv GH, Zhang QS, Wang SF, Li J, Wang XB. (2018). Prognostic factors in skull base chordoma: a systematic literature review and meta-analysis. World Neurosurg.

